# Deficiency of the sphingosine-1-phosphate (S1P) transporter Mfsd2b protects the heart against hypertension-induced cardiac remodeling by suppressing the L-type-Ca^2+^ channel

**DOI:** 10.1007/s00395-024-01073-x

**Published:** 2024-08-07

**Authors:** Dragos Andrei Duse, Nathalie Hannelore Schröder, Tanu Srivastava, Marcel Benkhoff, Jens Vogt, Melissa Kim Nowak, Florian Funk, Nina Semleit, Philipp Wollnitzke, Ralf Erkens, Sebastian Kötter, Sven Günther Meuth, Petra Keul, Webster Santos, Amin Polzin, Malte Kelm, Martina Krüger, Joachim Schmitt, Bodo Levkau

**Affiliations:** 1 Institute for Molecular Medicine III, University Hospital Düsseldorf and Heinrich Heine University, Düsseldorf, Germany; 2 Department of Cardiology, Pneumology, and Vascular Medicine, Medical Faculty, Heinrich Heine University, Düsseldorf, Germany; 3 Cardiovascular Research Institute Düsseldorf (CARID), Düsseldorf, Germany; 4 Institute of Pharmacology, University Hospital Düsseldorf, Düsseldorf, Germany; 5 Institute of Cardiovascular Physiology, Medical Faculty and University Hospital Düsseldorf, Heinrich-Heine-University Düsseldorf, Düsseldorf, Germany; 6 Department of Neurology, Medical Faculty, Heinrich Heine University of Düsseldorf, Düsseldorf, Germany; 7 Department of Chemistry and Virginia Tech Center for Drug Discovery, Virginia Tech, Blacksburg, VA 24060 USA

**Keywords:** Sphingosine-1-phosphate, Left-ventricular remodeling, Cardioprotection, Mfsd2b

## Abstract

The erythrocyte S1P transporter Mfsd2b is also expressed in the heart. We hypothesized that S1P transport by Mfsd2b is involved in cardiac function. Hypertension-induced cardiac remodeling was induced by 4-weeks Angiotensin II (AngII) administration and assessed by echocardiography. Ca^2+^ transients and sarcomere shortening were examined in adult cardiomyocytes (ACM) from Mfsd2b^+/+^ and Mfsd2b^−/−^ mice. Tension and force development were measured in skinned cardiac fibers. Myocardial gene expression was determined by real-time PCR, Protein Phosphatase 2A (PP2A) by enzymatic assay, and S1P by LC/MS, respectively. Msfd2b was expressed in the murine and human heart, and its deficiency led to higher cardiac S1P. Mfsd2b^−/−^ mice had regular basal cardiac function but were protected against AngII-induced deterioration of left-ventricular function as evidenced by ~ 30% better stroke volume and cardiac index, and preserved ejection fraction despite similar increases in blood pressure. Mfsd2b^−/−^ ACM exhibited attenuated Ca^2+^ mobilization in response to isoprenaline whereas contractility was unchanged. Mfsd2b^−/−^ ACM showed no changes in proteins responsible for Ca^2+^ homeostasis, and skinned cardiac fibers exhibited reduced passive tension generation with preserved contractility. Verapamil abolished the differences in Ca^2+^ mobilization between Mfsd2b^+/+^ and Mfsd2b^−/−^ ACM suggesting that S1P inhibits L-type-Ca^2+^ channels (LTCC). In agreement, intracellular S1P activated the inhibitory LTCC phosphatase PP2A in ACM and PP2A activity was increased in Mfsd2b^−/−^ hearts. We suggest that myocardial S1P protects from hypertension-induced left-ventricular remodeling by inhibiting LTCC through PP2A activation. Pharmacologic inhibition of Mfsd2b may thus offer a novel approach to heart failure.

**Supplementary Information:**

The online version contains supplementary material available at 10.1007/s00395-024-01073-x.

## Introduction

Hypertension represents a major risk factor for the development of cardiovascular diseases [5] affecting more than 1 billion people worldwide [40, 41]. In hypertensive patients, the heart is subjected to chronic pressure overload and adapts by left-ventricular hypertrophy (LVH) [18, 34], with a high prevalence of LVH in hypertensive populations [3]. Accompanied by overactivation of the renin–angiotensin–aldosterone axis, sustained hypertension leads to heart failure [5, 17, 57] and, subsequently, increased mortality [34]. Cardiomyocyte calcium (Ca^2+^) overload is a major factor predisposing the myocardium to heart failure [49]. Physiologically, myocardial Ca^2+^ homeostasis controls the balance between relaxation and contraction in the cardiac systole and diastole [7, 13]. This homeostasis is controlled by voltage-gated L-type calcium channels (LTCC), the sodium-calcium exchanger (NCX) and the SR calcium ATPase (SERCA2a) [8].

Sphingosine-1-phosphate (S1P) is a bioactive sphingolipid with numerous effects on the cardiovascular system, where it acts both as an intracellular second messenger and extracellular ligand to five G-protein coupled receptors (S1PR1-5) [24, 32, 54]. In the heart, S1P modulates contractility [20, 35], Na^2+^ and Ca^2+^ homeostasis [25], protects against ischemia/ reperfusion (I/R) injury [10, 51, 60], improves cardiac remodeling after I/R [45], contributes to preconditioning [19, 24, 25, 61] and participates in the pathophysiology of hypertrophy and heart failure [47]. Plasma S1P levels in humans have been linked to hypertension [21], heart failure [63], myocardial infarction [50], and ischemic heart diseases [44].

Plasma S1P is mainly supplied by endothelial cells (EC), red blood cells (RBC), and platelets through two designated S1P transporters: Spinster-2 (Spns2) in EC [11] and Major facilitator superfamily domain containing 2 b (Mfsd2b) in RBC and platelets, respectively [62]. We have recently shown that Mfsd2b-dependent release of S1P from platelets limits infarct size during murine myocardial infarction, and that plasma S1P levels after myocardial infarction are associated with reduced cardiovascular mortality in ST-elevation myocardial infarction patients [42]. In the present study, we asked the question if Mfsd2b is expressed in the adult heart and, if so, whether it plays a functional role in an angiotensin II (AngII) model of heart failure.

## Methods

### Mouse models

All mouse experiments were approved by the local animal ethics committee (LANUV Recklinghausen, Germany) under file number: 81-02.04.2021.A012 and performed according to the European Convention for the Protection of Vertebrate Animals Used for Experimental and Other Scientific Purposes (Council of Europe Treaty Series No. 123). Mouse care was executed according to the institutional guidelines. Mice were housed within the central animal research facility of the Heinrich Heine University Düsseldorf on a 12/12 h light/dark cycle with ad libitum drinking water and a standard rodent diet. Experiments using mice were planned according to the ARRIVE recommendations [26]. Mfsd2b^–/–^ mice were purchased from the MMRRC at UC Davis. All experiments were performed in 22 week old male mice.

### Hypertension-induced cardiac remodeling

Global Mfsd2b^−/−^ and Mfsd2b^+/+^ mice were subjected to 4-weeks AngII (1000 ng·kg^−1^·min^−1^) treatment using subcutaneous osmotic minipumps (model 2004, Alzet, Cupertino, CA). Before implantation, mice were anesthetized with isoflurane (2–2.5% initiation, 1.5–2% steady state). This induced reliably hypertension with consecutive cardiac remodeling and left-ventricular deterioration as previously shown [56]. Following the 4-week treatment and after the echocardiographic assessment of left-ventricular functional parameters, mice were sacrificed, and hearts collected for further analysis.

### Echocardiographic assessment of cardiac function

Cardiac echocardiography was performed before and after AngII treatment as described [9, 43, 45]. Mice were anesthetized using isoflurane anesthesia (2–2.5% initiation, 1.5–2% steady state) with constant cardiocirculatory rate (heart rate: 400–600 bpm, breathing rate: ~ 100/min). The left-ventricular systole and diastole were recorded using a high-resolution ultrasound system (18–38 MHz; Vevo 3100, Visual Sonics Inc., Toronto, Canada). Functional parameters, i.e., left-ventricular volume at the end of systole or diastole, stroke volume, cardiac output, and ejection fraction, were calculated in B-mode by identification of maximal and minimal cross-sectional area using the manufacturer’s software Vevo Lab 5.6.1 (Visual Sonics Inc., Toronto, Canada).

### Liquid chromatography with tandem mass spectrometry (LC‐MS/MS)

Plasma and tissue samples were extracted by precipitation in methanol. Internal standard (1 µM C17 S1P in MeOH, 10 µl) was added to weighted heart tissue, homogenized in MeOH (1 ml), and precipitated at − 80 °C overnight. Tissue sample supernatants were concentrated in a vacuum rotator (60 °C, 1 h), the residue dissolved in MeOH (100 µL) and transferred into mass spectrometry vials. Chromatographic separation was performed on an LCMS-8050 triple-quadrupole mass spectrometer (Shimadzu Duisburg, Germany) interfaced with a Dual Ion Source and a Nexera X3 Front-End-System (Shimadzu Duisburg, Germany). High-performance liquid chromatography was performed with a 60 × 2 mm MultoHigh-C18 RP column with 3 µm particle size at 40 °C (CS Chromatographie Service, Langerwehe, Germany). Mobile phases consisted of [A] = MeOH and [B] = 1% (v/v) aq. HCO2H. The following LC gradient settings were used: Nebulizer: 3 L/min, Interface Temperature 300 °C, Desolvation Temperature 526 °C, Heat Block Temperature 400 °C and Drying Gas Flow 10 L/min. The flow rate was 400 µL/min, and injection volume 10 µL (Supplemental Table 1). Standard curves were generated by measuring increasing amounts (10 fmol to 50 pmol) of S1P and internal standard (C17 S1P, 0.1 µM final conc. in MeOH). The linearity of the standard curves and correlation coefficients were obtained by linear regression analysis. Data were collected using multiple reaction monitoring (MRM). The mass transitions for analysis in multiple reaction monitoring (MRM) modes were m/z = 380/264 and 380/82 for S1P and *m*/*z* = 366/250 for C17 S1P. Data analysis was performed with LabSolutions 5.114 (Shimadzu, Kyoto, Japan).

### Western blotting

Isolated ACMs were preincubated with sphingosine (1 µM) for 30 min and stimulated with isoprenaline (1 µM) for 5 min. Cells were lysed in RIPA buffer (in mM: 10 Tris pH 8.0, 0.5 EGTA, 0.5% Triton™ X-100, 0.1% desoxycholic acid, 0.1% SDS, 140 NaCl) containing protease inhibitors and phosphatase inhibitors (HALT™, Thermo Scientific). Protein concentration was determined by BCA assay (Pierce) and 50 µg of lysate mixed with 4 × Laemmli sample buffer without boiling. Samples were separated on a 6% gel and transferred to a PVDF membrane (400 mA, 4 h, on ice). The membranes were probed for p-LTCC-Ser1981 (PA5-64748, 1:1000) and Calsequestrin/CASQ (26,625, 1:1000) both from Thermo Fisher, Germany. Heart tissue was snap-frozen and stored at − 80 °C. Lysates were prepared Heart by mechanical disruption (Ultra-Turrax^®^ T10 Basic, Ika^®^) in modified RIPA buffer containing protease and phosphatase inhibitors before adding Laemmli sample buffer. To preserve phospholamban (PLN) oligomers, samples were only heated to 40 °C. Protein concentrations were determined by bicinchoninic acid assay (Pierce^®^ BCA Assay Kit, Thermo Scientific) and equal amounts of total protein were loaded to the gels. Proteins were separated by SDS-PAGE with 15% polyacrylamide gels (6% for RyR2) and transferred to polyvinylidene difluoride (PVDF) membrane (Immobilon-P, 0.45 µm pore size, Millipore). For titin analysis frozen heart tissue was solubilized in modified Laemmli buffer and proteins were separated on agarose-strengthened 2.1% PAGE. To determine titin isoform composition proteins were visualized by imperial protein staining solution (Thermo Scientific). To analyze titin phosphorylation proteins were then transferred to a PVDF -membrane and processed as previously described [28]. Membranes were blocked with 0.5% milk/TBS-T (in mM: 10 Tris, 150 NaCl, 0.1% Tween 20, and pH 7.6, SNAP i.d.^®^ 2.0, Millipore) and incubated with primary antibodies overnight at 4 °C in 5% milk/TBS-T. Reactive bands were visualized by enhanced chemiluminescence using horse-radish peroxidase-coupled secondary antibodies (abcam) and Immobilon^®^ Forte Western HRP substrate (Millipore). Signals were digitally quantified using a CCD camera system and associated software (ChemStudio, VisionWorks, AnalytikJena). The following antibodies were used for detection: Ryanodine receptor 2 (RyR2) (Thermo Fisher Scientific, PA5-87,416, 1:5000 dilution), pRyR2(S2814) (Badrilla, S2814, A010-31, 1,5000), PLN (Badrilla, clone A1, 1:5000), phospho-PLN(S16) (Badrilla, S16, 1:5000), cardiac Troponin I (TnI) (Cell Signaling Technology®, #4002, 1:5000), phospho-TnI (S23/24) (Cell Signaling Technology®, #4004, 1:5000), Troponin T (TnT) (S23/24) (Cell Signaling Technology®, #5593, 1:5000), Calsequestrin (PA1–913, Thermo Fisher Scientific, 1:5000 dilution), cardiac-type myosin-binding protein C (MYBPC3) (Thermo Fisher Scientific, #703574, 1:5000), myosin light chain 2 (MLC2) (Affbiotech, 1:5000), phospho-MLC2 (S15) (Affbiotech, 1:5000), α-PKCα (Abcam, ab32376), α-phospho-PKCα T497 (Abcam, ab76016). Titin domain phosphorylation was tested using custom made (Eurogentec) phosphosite directed antibodies to pSer4010, pSer4099, pSer4062, and pSer11878 (human cardiac titin; UniProtKB: Q8WZ42) and control antibodies recognizing the phosphorylated and unphosphorylated PEVK-fragment (pan-Titin). Chemiluminescent detection using horse-radish peroxidase-coupled secondary antibodies (Sigma) and Immobilon Forte HRP substrate (Millipore) was used to visualize signals. Quantitative analysis of unsaturated signals in the linear range of detection was performed using Fusion SOLO imaging system (Vilber Lourmat) and associated software (Multi Gauge V3.2 or ChemStudio, AnalytikJena). Differences in phosphorylation levels, the ratio of signal intensities phospho:pan was calculated for each sample and with each set of phosphosite-directed antibodies.

### Blood pressure measurements by tail-cuff system

Blood pressure was measured noninvasively using a computerized tail-cuff system (Hatteras Inc.). Mice were subjected to several measurements before pump implantation to adapt to the device. After baseline measurements before pump implantation, blood pressure was measured in a 3-day interval for 25 days.

### Measurements of cardiomyocyte Ca^2+^ cycling and sarcomere length

Ca^2+^-cycling analyses were performed in isolated cardiomyocytes from Mfsd2b^+/+^ and Mfsd2b^−/−^ mice. For isolation, the murine hearts were digested by 6 min of retrograde perfusion with collagenase type I (Worthington Biochemical Corp., Lakewood, NJ, USA). The further procedures (i.e., ACM isolation, recalcification, cell loading with Fura-2 AM, and consecutive measurements) were performed similarly to those previously described [29, 52]. Briefly, after Fura-2 loading, the myocytes received superfusion with a 37 °C buffer containing (in mM) 137 NaCl, 5.4 KCl, 1.2 CaCl2, 1 MgCl2, 10 HEPES, 5.5 glucose, the pH of which was corrected to 7.4. ACM were then paced at 0.5 Hz in an electric field. Ca^2+^ transients were recorded at 340 and 380 nm excitation wavelengths using a dual-excitation fluorescence imaging/ contractility recording system (HyperSwitch Myocyte System, IonOptix Corp., Milton, MA, USA). Perfusion buffer containing 10 − 7 M isoprenaline (iso) was applied to the bath with Fura-2-loaded myocytes for β-adrenergic stimulation. Isoprenaline-stimulated cells were further measured upon exposure to the LTCC inhibitor verapamil (10^−5^ M). Data analysis was performed using IonWizard software (Version 6.4, IonOptix Corp.). Ratiometric data obtained at 340/380 nm in 10 independent Ca^2+^ transient measurements per myocyte were averaged. One heart composed readings from 10 corresponding myocytes, and 10 hearts were analyzed per group. Sarcomere contraction and relaxation variables were measured in paced cardiomyocytes via video detection (MyoCam-S™, IonOptix). Data were analyzed using IonWizard software (Version 6.4).

### Preparation of skinned cardiac fibers and mechanical measurements

Papillary muscles were prepared from the left ventricles of Mfsd2b^+/+^ and Mfsd2b^−/−^ mice and stored at − 20 °C in 50% glycerol: 50% low ionic strength buffer (75 mM potassium chloride, 10 mM Tris–HCl, 2 mM Mg-chloride, 2 mM EGTA, 100 mM protease inhibitor cocktail, 50 mM phosphatase inhibitor cocktail, pH 7.1) until use. For tension measurements ,papillary muscles were skinned overnight in relaxing solution (7.8 mM ATP, 20 mM creatine phosphate, 20 mM imidazole, 4 mM EGTA, 12 mM Mg-propionate, 97.6 mM K-propionate, pH 7.0, 30 mM 2,3-butanedione monoxime (BDM), 1 mM dithiothreitol (DTT), 100 mM protease inhibitor cocktail, 50 mM phosphatase inhibitor cocktail) supplemented with 1% wt/vol TritonX-100 on ice. The skinned tissues were then extensively washed in relaxing solution without TritonX-100, and small fiber bundles with diameters of 200–350 μm and a length of 1.0–2.0 mm were dissected. Force measurements were performed with a muscle mechanics workstation (Myotronic, Heidelberg, Germany) at room temperature [30]. Skinned left-ventricular fiber bundles were bathed in relaxing solution (7.8 mM ATP, 20 mM creatine phosphate, 20 mM imidazole, 4 mM EGTA, 12 mM Mg-propionate, 97.6 mM K-propionate, pH 7.0, 30 mM BDM, 1 mM DTT, 100 mM protease inhibitor cocktail, 50 mM phosphatase inhibitor cocktail) and mounted to the motor arm and force transducer between stainless steel clips. Passive tension was determined by stepwise stretching the mounted fibers to a maximum of 130% of slack length. To measure Ca^2+^-sensitivity of force development, skinned fiber bundles were mounted in relaxing solution without BDM and were pre-stretched by 20% of their slack length. Force-pCa relations were determined by sequentially increasing Ca^2+^-concentration to pCa 4.5. Averaged data (mean ± SEM) on relative-force vs. pCa diagrams were fitted by using the Hill equation. Force data were related to cross-sectional area determined from the diameter of the specimens (by assuming a cylindrical shape and circular cross-sectional area).

### Quantitative PCR

RNA was isolated from heart tissue and ACM using the innuPREP RNA Mini Kit (Analytik Jena, Jena, Germany) according to the manufacturer instructions. Synthesis of cDNA (200 ng) was performed by using the Revert Aid First Strand cDNA Synthesis Kit (Thermo Fisher Scientific, Waltham, USA). Gene expression was quantified by quantitative PCR (qPCR) via SYBR Green (Bio-Rad Laboratories, Hercules, USA). Primer sequences are listed in Supplemental Table 2. As an internal control for normalization GAPDH was used.

### Histology

Heart tissue was fixed in 4% paraformaldehyde overnight and embedded in paraffin. Serial transverse sections of 5 µM were stained with Sirius red.

### PP2A assay

PP2A activity in heart tissue of Mfsd2b^+/+^ and Mfsd2b^−/−^ mice was assessed using the PP2A Immunoprecipitation Phosphatase Assay Kit (# 17-313 from Merck, Darmstadt, Germany) with minor modifications to the manual’s procedure To immunoprecipitate PP2A, heart tissue lysates containing 1000 µg protein or murine ACM containing 500 µg protein were incubated in a total volume of 500 µl pNPP Ser/Thr Assay buffer with 8 µg of anti-PP2A-C subunit antibody (clone 1D6, #05-421) and 25 µl of protein A agarose slurry for 18 h at 4 °C with constant head over head rotation of 10 rpm. Phosphatase activity was determined via the amount of free inorganic phosphate released by the enzymatic conversion of threonine phosphopeptide. The generated orthophosphate reacts with the malachite green detection solution and is quantified by measuring absorption at 650 nm in a microplate reader (BMG LABTECH GmbH, Offenburg, Germany) after 35 min of development. PP2A activity was also measured in ACM cultured in M199 media (containing creatine 5 mM; carnitine 2 mM; taurine 5 mM;1 × Insulin-Transferrin-Selen/Anti-Anti/glutamine; 5% FCS, 10 µM blebbistatin) on laminin (10 µg/mL) stimulated for 30 min as indicated and harvested in lysis buffer with protease inhibitor on ice. For LC‐MS/MS measurements, 150,000 ACM were transferred to a laminin (10 µg/mL) coated cell culture plate and incubated with sphingosine (1 µM) or vehicle (ethanol) control for 30 min. After washing with ice-cold HBSS the final pellet was taken up in methanol for LC–MS/MS.

### Statistical analysis

The presented results are displayed as mean ± standard error of the mean (SEM). Unless otherwise specified, we used an unpaired Student’s *t* test (for normally distributed data) or a Mann–Whitney-*U*-Test (for non-normally distributed data) to test whether the two data groups were significantly different. Normal distribution was tested through the D’Agostino-Pearson test. For repeated measurements, data were analyzed by one-way and two-way analysis of variance (ANOVA), as appropriate, followed by Bonferroni’s, Sidak’s or Holm-Šídák’s multiple comparisons test post hoc tests. *p* < 0.05 was considered statistically significant. Statistical analysis was done using GraphPad Prism 9 for Windows (GraphPad Software, San Diego, CA, USA).

## Results

### Mfsd2b is expressed and functionally active in the murine and human heart

We observed that Mfsd2b is expressed in the adult C57BL/6 mouse heart and in isolated adult mouse cardiomyocytes by real-time qPCR (Suppl. Fig. 1A). S1P concentrations were 1.6-fold higher in Mfsd2b^−/−^ hearts compared to Mfsd2b^+/+^ hearts as determined by LC/MS–MS suggesting functional activity (Mfsd2b^+/+^: 1.19 ± 0.13 vs. Mfsd2b^−/−^: 1.91 ± 0.16 pmol/mg heart tissue; *p* = 0.0022). Mfsd2b was also expressed in human hearts (Suppl. Fig. 1B).

### Mfsd2b^−/−^ mice are protected against AngII-induced cardiac remodeling

**Fig. 1:**
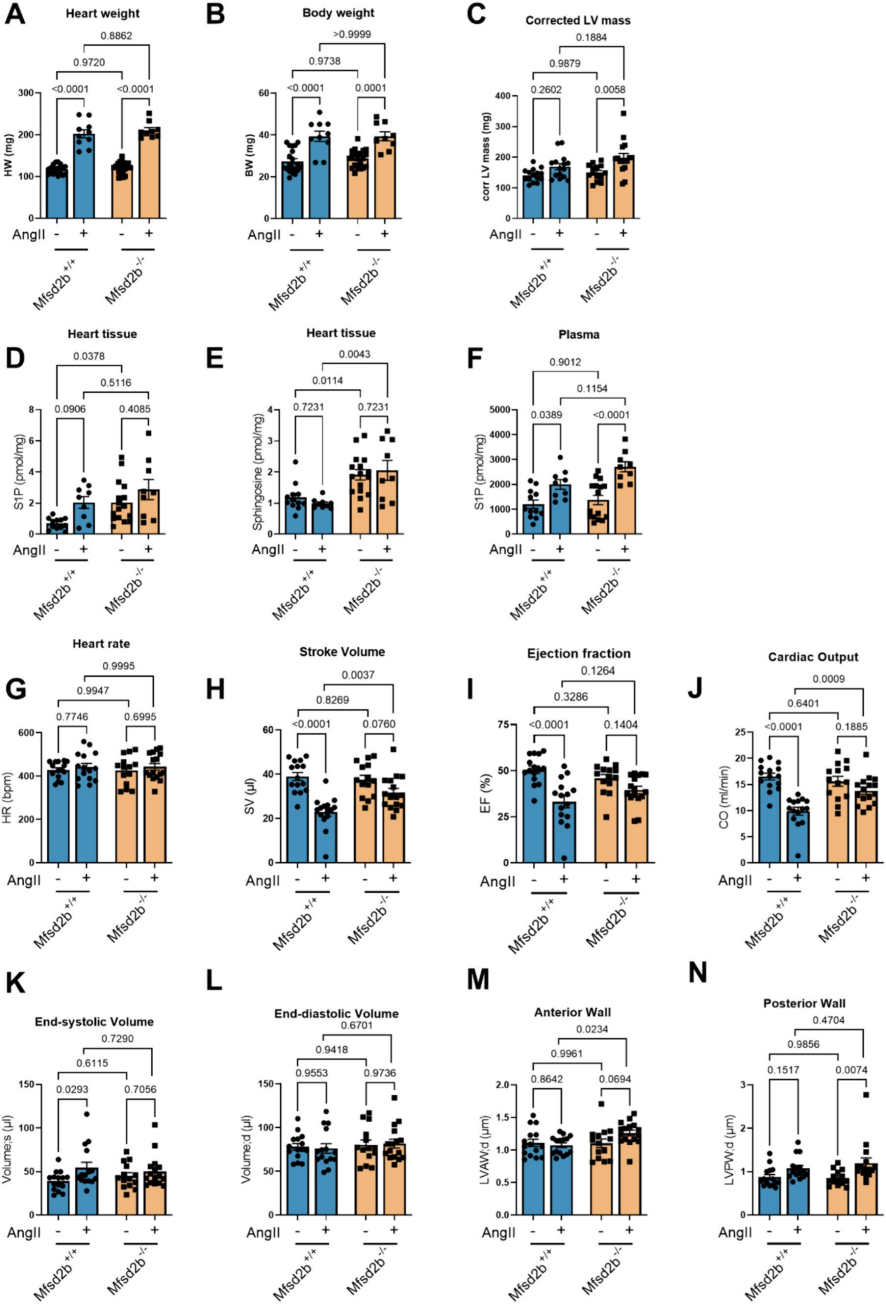
Four weeks of AngII infusion deteriorate the left-ventricular function of Mfsd2b^+/+^ but not Mfsd2b^−/−^ mice. Mfsd2b^−/−^ leads to cardiac S1P and Sphingosine accumulation. **A** Heart weights of Mfsd2b^+/+^ and Mfsd2b^−/−^ with and without AngII treatment. *n*_WT_ = 20, *n*_WT+AngII_ = 10, *n*_KO_ = 21, *n*_KO+AngII_ = 9. Data is presented as mean ± SEM. Statistical significance was analyzed by two-way ANOVA followed by Sidak’s multiple comparison test. *p* values are as indicated above the compared data sets. **B** Body weights of Mfsd2b^+/+^ and Mfsd2b^−/−^ before and after AngII treatment. *n*_WT_ = 20, *n*_WT+AngII_ = 10, *n*_KO_ = 21, *n*_KO+AngII_ = 9. Data is presented as mean ± SEM. Statistical significance was analyzed by two-way ANOVA followed by Sidak’s multiple comparison test. *p* values are as indicated above the compared data sets. **C** Corrected left-ventricular mass after AngII treatment measured by B-mode echocardiography. *n*_WT_ = 15, *n*_WT+AngII_ = 15, *n*_KO_ = 14, *n*_KO+AngII_ = 16. Data is presented as mean ± SEM. Statistical significance was assessed by RM two-way ANOVA followed by Sidak’s multiple comparison test. *p* values are as indicated above the compared data sets. **D** S1P and **E** Sphingosine levels in heart tissue from Mfsd2b^+/+^ and Mfsd2b^−/−^ mice before and after AngII treatment as measured by LC–MS/MS. *n*_WT_ = 12, *n*_WT+AngII_ = 9, *n*_KO_ = 16, *n*_KO+AngII_ = 9. Data is presented as mean ± SEM. Statistical significance was assessed by RM two-way ANOVA followed by Tukey’s multiple comparisons test. *p* values are as indicated above the compared data sets. **F** S1P levels in plasma from Mfsd2b^+/+^ and Mfsd2b^−/−^ mice before and after AngII treatment as measured by LC–MS/MS. *n*_WT_ = 12 *n*_WT+AngII_ = 9, *n*_KO_ = 16, *n*_KO+AngII_ = 9. Data is presented as mean ± SEM. Statistical significance was assessed by RM two-way ANOVA followed by Tukey’s multiple comparisons test. *p* values are as indicated above the compared data sets. **G** Heart rate, **H** Stroke volume, **I** left-ventricular ejection fraction, **J** cardiac output, **K** end-systolic volume, **L** end-diastolic volume, **M** anterior and **N** posterior wall thickness before and after 4-weeks of AngII treatment in the same mice. **G**–**N** *n*_WT_ = 15, *n*_WT+AngII_ = 15, *n*_KO_ = 14, *n*_KO+AngII_ = 16. Data is presented as mean ± SEM. Statistical significance was assessed by RM two-way ANOVA followed by Sidak’s multiple comparison test. *p* values are as indicated above the compared data sets

Heart weight and left-ventricular function were indistinguishable between Mfsd2b^−/−^ and Mfsd2b^+/+^ mice under basal conditions. However, AngII infusion for four weeks revealed clear differences in the cardiac response to pressure overload: Mfsd2b^+/+^ mice demonstrated intra-individual deterioration of left-ventricular function compared to baseline, whereas Mfsd2b^−/−^ mice were largely unaffected with preserved left-ventricular function. This was most evident in stroke volume (Mfsd2b^+/+^: 39 ± 1.8 at baseline vs. 22.9 ± 1.9 µl after AngII, *p* < 0.0001 compared to Mfsd2b^−/−^: 37.5 ± 2 at baseline vs. 31.7 ± 1.9 µl after AngII, *p* = 0.0760, Fig. 1H), ejection fraction (Mfsd2b^+/+^: 50.6 ± 2 at baseline vs. 33.2 ± 3.3% after AngII, *p* < 0.0001 compared to Mfsd2b^−/−^: 45.8 ± 2.2 at baseline vs. 39.5 ± 2.1% after AngII, *p* = 0.1404, Fig. 1I) and cardiac output (Mfsd2b^+/+^: 16.5 ± 0.7 at baseline vs. 9.9 ± 0.7 ml/min after AngII, *p* < 0.0001 compared to Mfsd2b^−/−^: 15.6 ± 0.9 (SEM) at baseline vs. 13.8 ± 0.7 ml/min after AngII, *p* = 0.1885, Fig. 1J). The end-systolic volume was also increased in Mfsd2b^+/+^ (39 ± 2.9 at baseline vs. 54.5 ± 6.1 µl after AngII, *p* = 0.0385), whereas it was unchanged in Mfsd2b^−/−^ (44.8 ± 3.8 at baseline vs. 50 ± 4.6 µl after AngII, *p* = 0.6767; Fig. 1K). The end-diastolic volumes and heart rates were unchanged between groups (Fig. 1G and L). Following AngII treatment, Mfsd2b^−/−^ showed a higher degree of hypertrophy, especially of the left-ventricular posterior wall (0.85 ± 0.05 at baseline vs. 1.2 ± 0.1 µl after AngII, *p* = 0.0074), which was not visible in Mfsd2b^+/+^ mice (0.87 ± 0.06 at baseline vs. 1.07 ± 0.07 µl after AngII, *p* = 0.1517, Fig. 1N). Blood pressure was similarly increased in both genotypes (Suppl. Fig. 2). Despite preserved left-ventricular function in Mfsd2b^−/−^ mice, the degree of hypertrophy after AngII was similar as measured by echocardiography, heart weight, fibrosis and embryonic gene expression (Fig. 1A–C and Suppl. Fig. 3). AngII treatment led to elevations in S1P and sphingosine levels in hearts of both strains (Fig. 1D, E) and resulted in higher plasma S1P (Fig. 1F) in both genotypes in line with the literature [36, 53].

### Mfsd2b^−/−^ cardiomyocytes display reduced Ca^2+^ mobilization and cycling but preserved contractility after isoprenaline

**Fig. 2:**
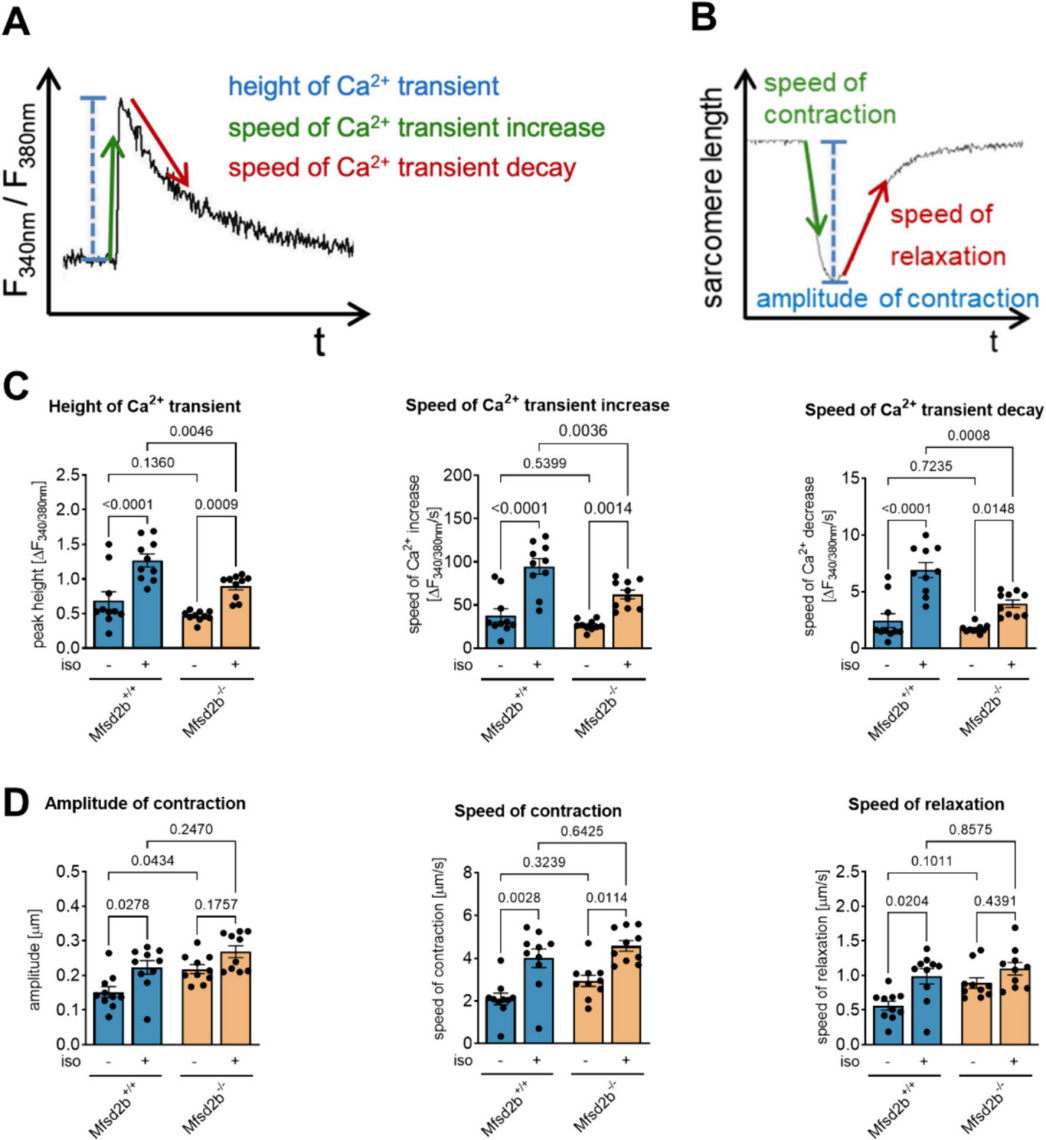
Impaired intracellular Ca^2+^ cycling but preserved sarcomere contractility of Mfsd2b^−/−^ ACM upon stimulation with isoprenaline. **A** Schematic representation of Ca^2+^ transients and **B** variables of sarcomere length. **B** Ca^2+^ transient amplitude and kinetics of paced Mfsd2b^+/+^ and Mfsd2b^−/−^ ACM at baseline and upon stimulation with isoprenaline (10^–7^ M). **C** Amplitude of contraction and kinetics of sarcomere shortening in Mfsd2b^+/+^ and Mfsd2b^−/−^ ACM at baseline and upon stimulation with isoprenaline (10^–7^ M). *n*_WT_ = 10, *n*_KO_ = 10. Statistical significance was assessed by two-way ANOVA followed by Tukey’s multiple comparisons test. *p* values are as indicated above the compared data sets

We assessed intracellular Ca^2+^ dynamics in ACM of Mfsd2b^+/+^ and Mfsd2b^−/−^ by measuring amplitude and speed of enrichment as well as speed of return to baseline (Fig. 2A and B). All parameters were similar under basal conditions between the genotypes but stimulation with isoprenaline revealed a clear ~ 50% reduction in Ca^2+^ amplitude, speed of enrichment and return to baseline, respectively, in ACM from Mfsd2b^−/−^ compared to Mfsd2b^+/+^ mice (Fig. 2C). Sarcomere contraction and relaxation patterns were similar under isoprenaline-stimulated conditions in both genotypes (Fig. 2D) as measured by contraction amplitude and contraction/relaxation speed. Of note, the amplitude of contraction was significantly higher in Mfsd2b^−/−^ cardiomyocytes under basal conditions (Fig. 2D). Comparisons between the two genotypes in non-parametric tests were also significant in speed of contraction and relaxation, respectively. This suggests that several myofilament proteins are relatively more sensitive to calcium-dependent activation resulting in similar contractility despite lower calcium cycling.

**Fig. 3:**
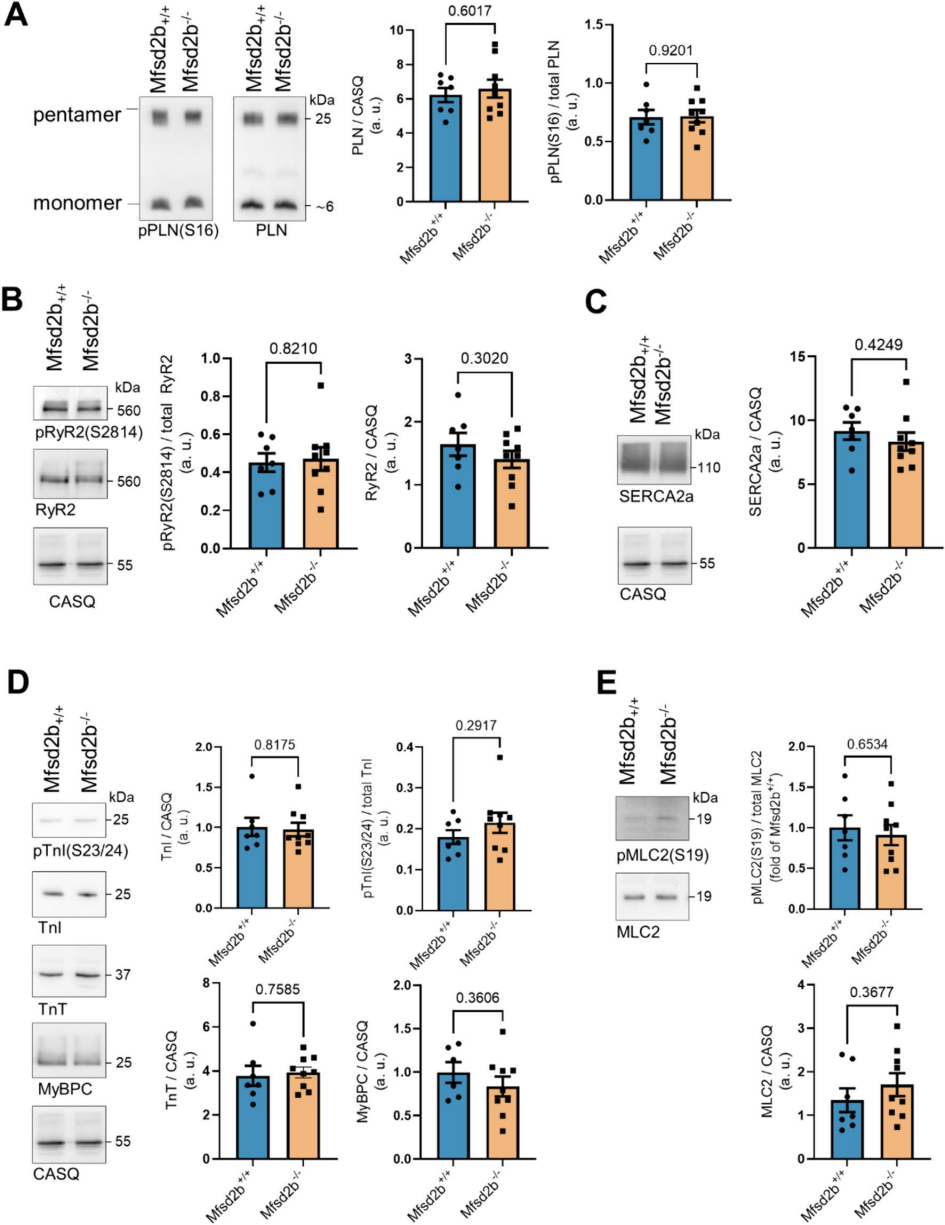
Similar phosphorylation levels of RyR2, PLN, MLC, SERCA2a, and TnI in heart tissue from Mfsd2b^+/+^ and Mfsd2b^−/−^ mice. WB analysis of phospho- and total-RyR2, PLN, MLC, SERCA2a, and TnI in the two genotypes. *n*_WT_ = 7, *n*_KO_ = 9. Data is presented as mean ± SEM. An unpaired Student *t* test was used for statistical analysis. *p* values are as indicated above the compared data sets

Analysis of expression and phosphorylation levels of the essential proteins responsible for Ca^2+^ homeostasis in Mfsd2b^−/−^ and Mfsd2b^+/+^ ACM revealed similar levels of p(S16)-PLN (Fig. 3A), p(S2814)-Ryr2 (Fig. 3B), and p(S19)-MLC2 (Fig. 3E) and no differences in the phosphorylation of the calcium-sensitive contractile protein TnI as determined by p(S23/24)-TnI levels (Fig. 3D). The total levels of RyR2, PLN, SERCA2a, MLC2, TNI, TNT, and MyBPC were also comparable between ACM of both genotypes (Fig. 3).

### Verapamil abolishes the differences in calcium cycling between Mfsd2b^+/+^ and Mfsd2b^−/−^

**Fig. 4:**
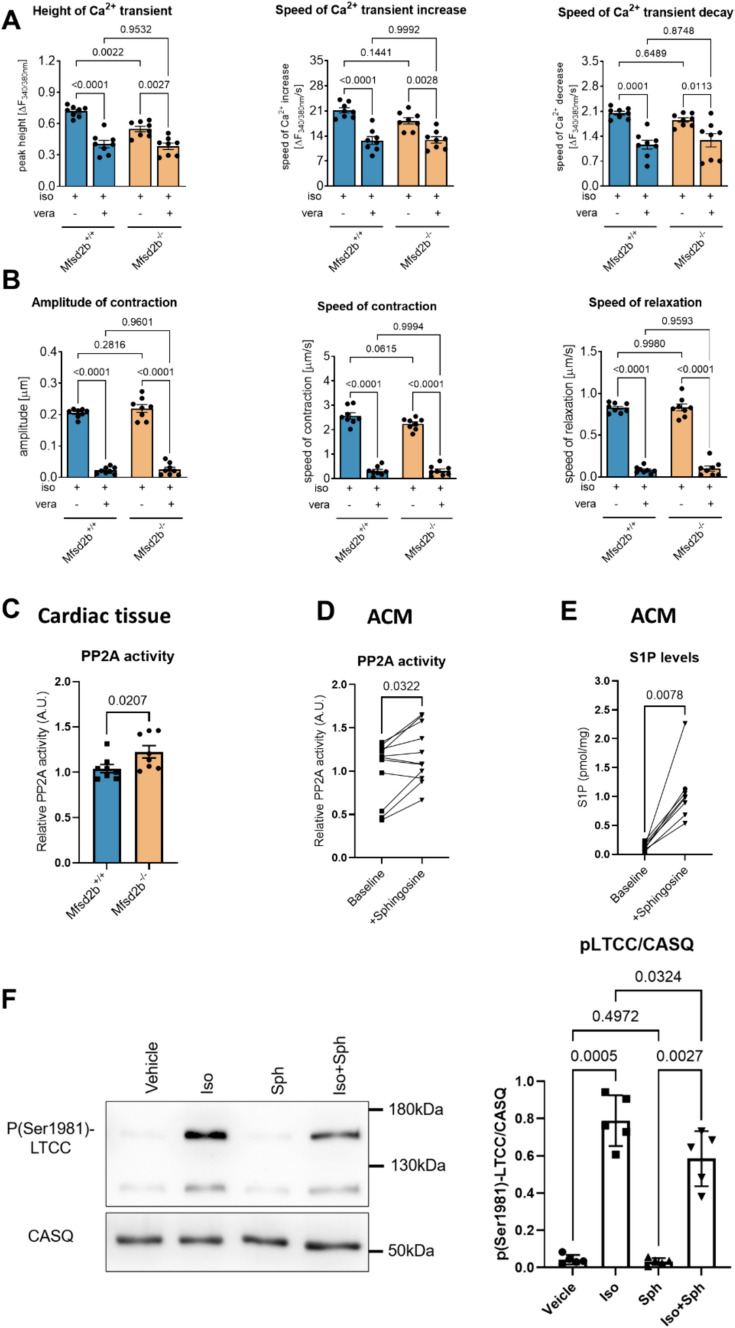
Reduced intracellular Ca^2+^ cycling upon stimulation with isoprenaline in Mfsd2b^−/−^ ACM is abolished by verapamil. Effects of Mfsd2b and S1P on PP2A and LTCC activity. **A** The LTCC inhibitor verapamil (vera, 10^–5^ M) abolished the reduced Ca^2+^ cycling of isoprenaline-stimulated Mfsd2b^−/−^ ACM. **B** Verapamil (vera, 10^–5^ M) effects on sarcomere contraction and relaxation in isoprenaline-treated ACM. *n*_WT_ = 8, *n*_KO_ = 8. Data is presented as mean ± SEM. Two-way ANOVA followed by Tukey’s multiple comparison test were used for statistical analysis. *p* values are as indicated above the compared data sets. **C** PP2A activity from cardiac tissue from Mfsd2b^+/+^ and Mfsd2b^−/−^ mice. *n*_WT_ = 8, *n*_KO_ = 8. Data is presented as mean ± SEM. An unpaired Students *t* test was used for statistical analysis. *p*-value is as indicated above the compared data sets. **D** PP2A activity in C57BL/6 ACM before and after S1P loading using sphingosine. *n* = 11 for both groups. A Wilcoxon test was used for statistical analysis *p*-value is as indicated above the compared data sets. **E** S1P levels in C57BL/6 ACM before and after S1P loading using sphingosine as measured by LC–MS/MS. *n* = 8 for both groups. A Wilcoxon test was used for statistical analysis. *p*-value is as indicated above the compared data sets. **F** Representative western blots and densitometric analysis of the levels of p(Ser1981)-LTCC and total LTCC in ACM from C57BL/6 at baseline and after stimulation with Isoprenaline (iso) (1 µM), Sphingosine (Sph) (1 µM) or both substances (Iso + Sph). *n*_Control_ = 5, *n*_Sph_ = 5, *n*_Iso_ = 5, *n*_Iso+Sph_ = 5. Statistical significance was analyzed by one-way ANOVA with Holm-Šídák's multiple comparisons test. *p* values are as indicated above the compared data sets

S1P has been shown to affect cardiomyocyte Ca^2+^ homeostasis and inhibit LTCC in rat ventricular myocytes [6, 25]. This prompted us to examine whether enhanced LTCC activity through S1P accumulation due to lack of Mfsd2b-mediated S1P export was responsible for the effects. Indeed, the marked differences in Ca^2+^ transients after isoprenaline stimulation between Mfsd2b^+/+^ and Mfsd2b^−/−^ were abolished in the presence of the LTCC blocker verapamil (Fig. 4A) and contractility suppressed to a similar extent (Fig. 4B).

### Increased PP2A activity in Mfsd2b^−/−^ whole hearts and cardiomyocytes

S1P inhibition of the L-type calcium current has been suggested to occur through PP2A signaling [6] but the mechanism has remained unexplored. PP2A was shown to regulate calcium transients by inhibiting protein kinase A-dependent LTCC activation by dephosphorylation [31]. Sphingoid bases and analoga such as sphingosine and C2 ceramide have been shown to disrupt membrane trafficking of glucose and amino acid transporters by activating PP2A [14, 22, 27], and that intracellular S1P directly binds to and activates the catalytic subunit of PP2A [58]. Thus, we measured PP2A activity in Mfsd2b^−/−^ and Mfsd2b^+/+^ hearts and observed it to be 1.2-fold higher in Mfsd2b^−/−^ compared to Mfsd2b^+/+^ (*p* = 0.0207, Fig. 4C). We then isolated ACM and showed that increasing intracellular S1P by supplementing sphingosine resulted in an eightfold increase of ACM S1P levels (0.13 ± 0.02 at baseline vs. 1.07 ± 0.18 pmol/mg.; *p* = 0.0078) and to a ~ 20% higher PP2A activity (338.4 ± 35.5 at baseline vs. 410.5 ± 34.1 a.u.; *p* = 0.0322) (Fig. 4D). This suggests that accumulation of S1P in cardiomyocytes leads to PP2A activation. We then stimulated ACM with isoprenaline after raising intracellular S1P (Fig. 4E) and measured Ser1981 phosphorylation in LTCC, a target site for PP2A [4, 16], as a proxy for LTCC activity. Five minutes of isoprenaline lead to a ~ 20-fold increase in Ser1981-LTCC phosphorylation that was reduced by ~ 25% in S1P-loaded cells (Fig. 4F). This is a clear indication that raising intracellular S1P inhibits LTCC phosphorylation and hence activity.

### Cardiac fibers from Mfsd2b^−/−^ mice show reduced passive tension generation upon stretch but preserved contractile function

**Fig. 5:**
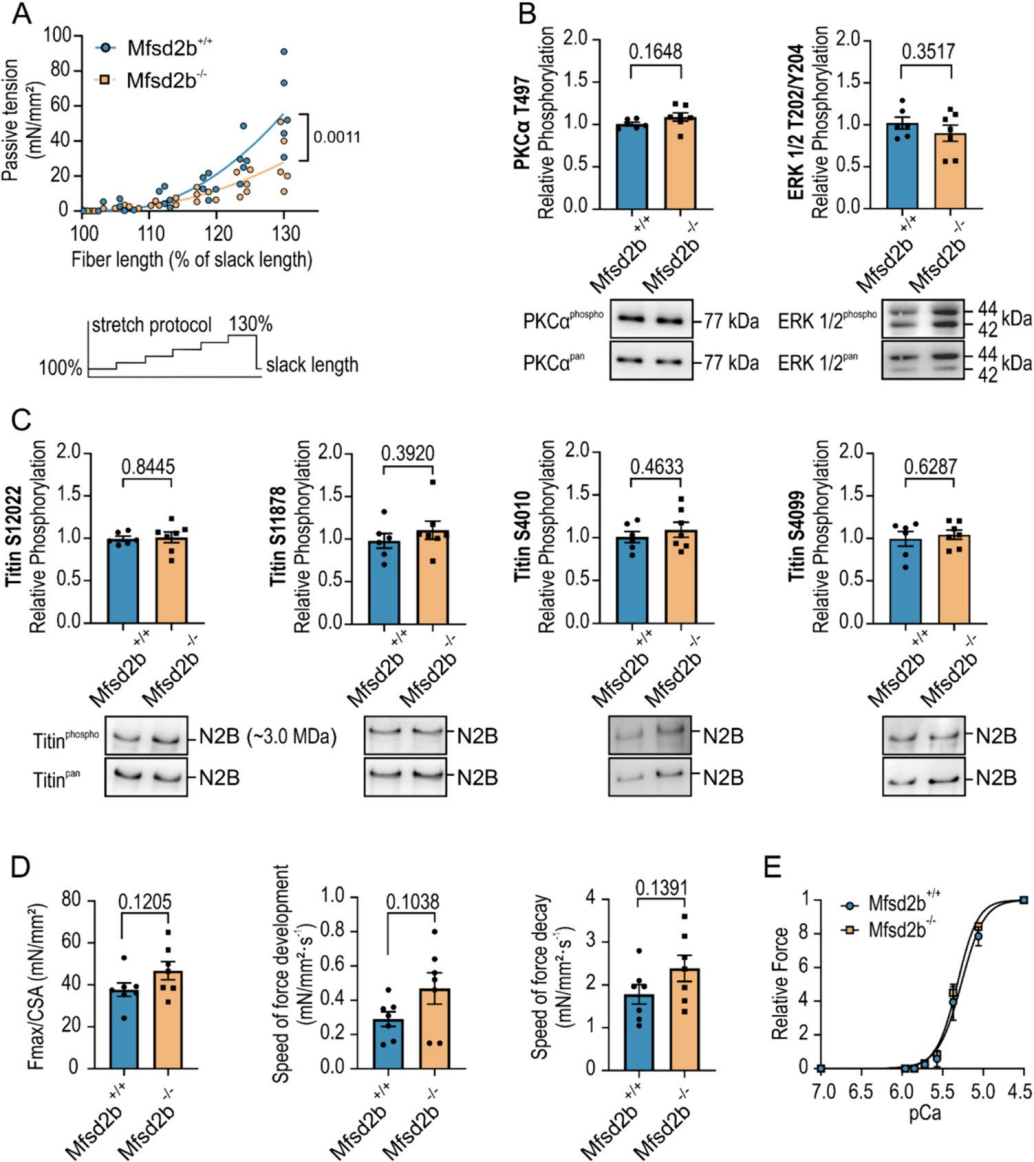
Reduced passive tension but preserved Ca^2+^-dependent force production in cardiac tissue from Mfsd2b^−/−^ mice. **A** Individual data points comparing passive tension values of permeabilized fiber bundles from papillary muscle after stepwise stretching to 110, 120 or 130% of resting fiber length. Lines represent polynomial fit to the data. Schematic stretch protocol is shown below the graph. **B** Western blot analyses of left-ventricular tissue from Mfsd2b^+/+^ and Mfsd2b^−/−^ hearts using phospho-specific antibodies to PKCα T497 and ERK 1/2 T202/Y204. Signals were normalized to total PKCα or ERK 1/2. Representative Western blot images are shown below the bar graphs. **C** Western blot analyses using phospho-specific antibodies to titin phosphorylated at S12022, S11878, S4010 and S4099. Signals were normalized to total titin. Bar graphs show mean ± SEM from *n*_WT_ = 6 and *n*_KO_ = 7 mice. **D** Maximum Ca^2+^-induced force development at pCa 4.5 (Fmax) and speed of force development and force decay. Bar graphs show mean ± SEM from *n*_WT_ = 7 and *n*_KO_ = 7 mice. **E** Assessment of Ca^2+^-sensitivity by measurement of Ca^2+^-dependent force in permeabilized papillary muscle strips from Mfsd2b^+/+^ and Mfsd2b^−/−^ hearts. Curves are Hill fits to the mean data points. Mechanical measurements were performed on *n*_WT_ = 11 and *n*_KO_ = 11. Data is shown as mean ± SEM. Statistical analysis of data in bar graphs was performed using unpaired Student’s *t* test, passive tension was analyzed using 2-way ANOVA with Sidak’s multiple comparisons test. *p* values are as indicated above the compared data sets

It is assumed that an increase in cardiomyocyte passive stiffness and calcium sensitivity can partially compensate for functional limitations caused by disturbed cellular Ca^2+^ handling [12]. We therefore tested passive tension using permeabilized fiber bundles from papillary muscles of Mfsd2b^+/+^ and Mfsd2b^−/−^ and detected a 40–45% reduction in the force-extension curve of fibers from Mfsd2b^−/−^ mice, when stretched to > 118% of slack length (Fig. 5A). Prompted by this finding we performed Western blot analyses using left-ventricular tissue samples from both groups and tested for phosphorylation (indicative for activation) of the kinases PKCα and ERK1/2 and of the sarcomeric target protein titin, a major determinant of cardiomyocyte passive stiffness. Our analyses showed no statistically significant difference in the relative phosphorylation of PKCα and ERK1/2, and of titin phosphorylation at S12022 and S11878 in the PEVK region of the protein, and at S4010 and S4099 in the N2B region (Fig. 5B, C).

Despite the clear reduction in Mfsd2b^−/−^ fibers’ passive tension upon stretch, maximal Ca^2+^ -induced force production was similar (37.7 ± 3.2 mN/mm^2^ in Mfsd2b^+/+^ and 46.7 ± 4.4 mN/mm^2^ in Mfsd2b^−/−^; *p* = 0.1205) as were kinetics of force development and force decay (0.29 ± 0.04 mN/mm^2^*s and 1.78 ± 0.23 mN/mm^2^*s in Mfsd2b^+/+^ vs. 0.47 ± 0.09 mN/mm^2^*s and 2.39 ± 0.30 mN/mm^2^*s in Mfsd2b^−/−^; Fig. 5D). Fibers from both genotypes displayed also similar Ca^2+^ -sensitivity of force development with log_EC_50 of 5.648 in Mfsd2b^+/+^ and 5.649 in Mfsd2b^−/−^ mice (Fig. 5E).

## Discussion

The current study presents the following novel findings: (1) The S1P exporter Mfsd2b, previously characterized only in RBC, is expressed and functional in the heart where it regulates cardiomyocyte S1P export; (2) Lack of Mfsd2b in cardiomyocytes and the concomitant increase in intracellular S1P suppress intracellular calcium cycling; (3) This is mediated by lower LTCC activity as measured by reduced Ser1981 phosphorylation due to S1P-mediated activation of its negative regulator PP2A; (4) This mechanism protects the heart against AngII-mediated cardiac remodeling.

S1P has multiple effects on cardiomyocyte function, ion currents and contractility but almost all of them are exerted through S1P receptor signaling [20, 24, 35, 61]. In contrast, we have identified a novel function of intracellular S1P in cardiomyocytes that became apparent when Mfsd2b was missing. Although intracellular effects of S1P as a second messenger have long been known [55] and were described even before the first S1P receptors were cloned, only few targets of intracellular S1P have been identified and none in relation to calcium. In fact, acute release of caged S1P by photolysis was shown to mobilize intracellular calcium from thapsigargin-sensitive stores independently of S1P receptor signaling some 15 years ago [39]. In contrast, chronically high intracellular S1P as in Mfsd2b deficiency attenuated isoprenaline-induced calcium mobilization in our hands but did not affect cardiomyocyte contractility. While the former suggests that calcium influx and/or release were affected, the latter implies that the calcium sensitivity of the contractile apparatus must have increased in a compensatory manner. In line with this, we have previously demonstrated higher calcium sensitivity of the contractile apparatus in cardiomyocytes after deletion of the S1P receptor 1 despite lower systolic and diastolic calcium [25]. In fact, extracellular and intracellular S1P signaling are interconnected as S1P receptor 1 has been shown to mobilize calcium by activating intracellular S1P production [38], hence the high intracellular S1P present in Mfsd2b-deficient cardiomyocytes may phenocopy S1P receptor 1 signaling. However, we did not find any changes in any proteins responsible for Ca^2+^ homeostasis and their phosphorylation in ACM. Furthermore, Ca^2+^ sensitivity of force development and phosphorylation of cardiac TnI were unchanged in permeabilized papillary muscle fibers from Mfsd2b^−/−^ mice. We hypothesized that increased passive stiffness of the contractile apparatus could contribute to the observed increased amplitude of contraction but found that papillary muscle fibers from Mfsd2b^−/−^ mice actually showed lower passive tension values in response to stretch. This reduction in passive tension was not explained by altered phosphorylation of titin tested at four different sites of phosphorylation in the elastic N2Bus and PEVK region, which have previously been described to modulate cardiomyocyte stiffness and affect myocardial function [33]. However, our results do not exclude the possibility that the reduction in passive tension is due to other post-translational modifications of titin or changes in the extracellular matrix (ECM). Several other proteins, such as the endothelial integrin-linked kinase and the cardiac fibroblast glycogen synthase kinase-3α, appear to be involved in cardiac function and its remodeling in diseases [46, 59]. Future studies should explore the compensatory mechanisms preserving contractility in Mfsd2b^−/−^ mice.

The pathophysiological consequence of our findings in a setting of hypertension-induced cardiac strain may be to protect the heart against deleterious calcium overload while preserving contractility. This would explain why Mfsd2b^−/−^ mice fared much better under AngII-induced cardiac remodeling in our study. In support of a more concentric type of hypertrophy taking place in Mfsd2b^−/−^ mice is the increased end-diastolic anterior and even more so posterior ventricular wall despite similar and rather small extent of fibrosis and identical increase in natriuretic peptide expression. This would also be in agreement with lower LTCC activity being causally involved as any differences in calcium kinetics between ACM of both genotypes were abolished in the presence of verapamil. Indeed, enhanced calcium influx through LTCC has been shown to be responsible for cardiac hypertrophy and pathologic remodelling, and LTCC blockers effectively inhibit cardiac remodelling under pressure overload [2, 37]. In line, phosphorylation of the PP2A target site Ser1981 in LTCC [4, 16] was clearly reduced by S1P-loading after isoprenaline stimulation. Finally, the phenotype of Mfsd2b^−/−^ was independent of blood pressure as both genotypes exhibited the same systolic blood pressure elevation with AngII.

We have previously shown that intracellular S1P directly activates PP2A in erythrocytes [58]. Among the myriad of physiological and pathophysiological processes PP2A regulates in cancer and diabetes, it also plays important roles in the heart, where it modulates calcium handling through its effects on ion channels, antiporters and pumps [31]. In particular, PP2A is a major negative regulator of LTCC activity and counteracts stimulation of voltage-gated calcium influx through LTCC after β-adrenergic stimulation [4]. In pacemaker cells, stimulation of PP2A by its upstream activator p21 activated kinase-1 (Pak1) repressed isoproterenol-stimulated LTCC activity [23]. Pak1 itself has been implicated in S1P-mediated inhibition of β-adrenergic stimulation of L-type calcium current [6] with intracellular S1P directly activating Pak 1 by a GTPase-independent mechanism thus bypassing its upstream regulatory proteins [1, 23]. Considering the abnormally high phosphorylation of LTCC and ryanodine receptors in heart failure, stimulation of PP2A activity has been suggested as a possible approach to restore the lost response to β-adrenergic stimulation in heart failure [31].

In the cancer field, small molecule activators of PP2A (SMAPs) as are currently being tested in clinical settings but must certainly be considered in cardiac diseases. Intriguingly, S1P analogs such as fingolimod (Gilenya^®^) have been known to inhibit PP2A quite some time through a mechanism involving displacement of PP2A’s own inhibitors SET and ANP32A [15, 48]. In contrast, we have recently shown S1P directly binds to activates the catalytic PP2A subunit in erythrocytes [58]. Here, we have clearly seen enhanced PP2A activity in Mfsd2b-deficient hearts in vivo and after increasing intracellular S1P in ACM cardiomyocytes in vitro. There was no immunosuppression in global Mfsd2b-deficient mice which would be an advantage over the use of PP2A activators such as fingolimod. Thus, pharmacologic Mfsd2b inhibitors may be a novel approach to activate PP2A indirectly through intracellular S1P accumulation and, importantly, without the immunosuppressive side effects of extracellular S1P receptor downregulation by fingolimod. However, other phosphatases that could be activated by S1P and dephosphorylate LTCC might exist. Currently, the first pharmacologic inhibitors of Mfsd2b are being developed, and any success in using them to treat heart failure will open a new avenue of drug research for cardiac diseases.

## Limitations

Although, Mfsd2b^−/−^ ACM exhibited a ~ 20% decrease in Ca^2+^ transients their sarcomere function was unchanged suggesting increased Ca^2+^ sensitivity of contractile proteins. However, neither did the calcium-sensitive proteins we looked at show any differences nor was muscle fiber force development altered. There was also no increase in cardiomyocyte passive stiffness (but rather a decrease instead) as another way cardiomyocytes employ to compensate for functional limitations caused by disturbed Ca^2+^ handling. We ruled out altered titin isoforms, ratios and post-translational modifications as reasons suggesting that other load-bearing cardiomyocyte structures such as actin, microtubules, and intermediate filaments might be altered in ways that influence cardiomyocyte passive stiffness. Outside of cardiomyocytes, ECM stiffness is known to determine the passive mechanical properties of muscle fibers. Whereas collagen content and cardiac fibrosis were unchanged in Mfsd2b^−/−^ hearts, collagen subtypes and/or crosslinks between ECM components that were not examined here may play a role. In addition, fibronectins, laminins and glycoproteins are also able to modify ECM stiffness. The exploration of the compensatory mechanisms that preserve contractility in Mfsd2b^−/−^ hearts is an exciting subject to be addressed in future studies.

## Supplementary Information

Below is the link to the electronic supplementary material.

Supplementary file1 (DOCX 1414 KB)

## Abbreviations

ACM: Adult cardiomyocytes
AngII: Angiotensin II
BDM: 2,3-Butanedione monoxime
Ca^2+^: Calcium
DTT: Dithiothreitol
EC: Endothelial cells
ECM: Extracellular matrix
I/R: Ischemia/reperfusion
KO: Knock-out
LC‐MS/MS: Liquid chromatography with tandem mass spectrometry
LTCC: L-type calcium channel
LVH: Left-ventricular hypertrophy
MeOH: Methanol
Mfsd2b: Major facilitator superfamily domain containing 2b
MLC2: Myosin light chain 2
MRM: Multiple reaction monitoring
MYBPC3: Myosin-binding protein C, cardiac-type
NCX: Sodium-calcium exchanger
Pak1: P21 activated kinase-1
PKCα: Protein kinase C alpha
PLN: Phospholamban
PP2A: Protein Phosphatase 2A
PVDF: Polyvinylidene difluoride
qPCR: Quantitative Polymerase Chain Reaction
RBC: Red blood cells
RyR2: Ryanodine receptor
SEM: Standard error of the mean
SERCA2a: Sarcoplasmic reticulum calcium ATPase
SMAPs: Small molecule activators of PP2A
Spns2: Spinster-2
S1P: Sphingosine-1-Phosphate
S1PR1-5: Sphingosine-1-Phosphate Receptors 1–5
TnI/T: Troponin I/T
