# Supplementary material for: Deficiency of the sphingosine-1-phosphate (S1P) transporter Mfsd2b protects the heart against hypertension-induced cardiac remodeling by suppressing the L-type-Ca2+ channel
Source: Basic Res Cardiol. 2024 Aug 7;119(5):853–68. doi: 10.1007/s00395-024-01073-x (PMC11461684; doi:10.1007/s00395-024-01073-x)
Supplement: Supplementary file 1 — Supplementary file1 (DOCX 1414 KB) [file 395_2024_1073_MOESM1_ESM.docx]

*Supplementary Information*

**Deficiency for the sphingosine-1-phosphate transporter Mfsd2b protects the heart against hypertension-induced cardiac remodeling by suppressing the L-type-Ca^2+^ channel**

Dragos Andrei Duse ^a,b,c^, Nathalie Hannelore Schröder ^a^, Tanu Srivastava ^d^, Marcel Benkhoff ^b^, Jens Vogt ^a^, Melissa Kim Nowak ^a^, Florian Funk ^d^, Nina Semleit ^a^, Philipp Wollnitzke ^a^, Ralf Erkens ^b,c^, Sebastian Kötter ^e^, Sven Günther Meuth ^f^, Petra Keul ^a^, Webster Santos ^g^, Amin Polzin ^b,c^, Malte Kelm ^b,c^, Martina Krüger ^c,e^, Joachim Schmitt ^c,d^, Bodo Levkau ^a,c^

^a^ Institute for Molecular Medicine III, University Hospital Düsseldorf and Heinrich Heine University, Düsseldorf, Germany.

^b^ Department of Cardiology, Pneumology, and Vascular Medicine, Medical Faculty, Heinrich Heine University, Düsseldorf, Germany.

^c^ Cardiovascular Research Institute Düsseldorf (CARID), Düsseldorf, Germany.

^d^ Institute of Pharmacology, University Hospital Düsseldorf, Düsseldorf, Germany.

^e^ Institute of Cardiovascular Physiology, Heinrich Heine University of Düsseldorf, Düsseldorf, Germany.

^f^ Department of Neurology, Heinrich Heine University of Düsseldorf, Düsseldorf, Germany.

^g^ Department of Chemistry and Virginia Tech Center for Drug Discovery, Virginia Tech, Blacksburg, VA 24060, United States.

Corresponding author:

Bodo Levkau, MD

Institute of Molecular Medicine III

University Hospital Düsseldorf

Heinrich Heine University, Düsseldorf, Germany

E-mail: bodo.levkau@med.uni-duesseldorf.de

Keywords (4/4-6): Sphingosine-1-phosphate, left-ventricular remodeling, cardioprotection, Mfsd2b.

**SI Fig. 1: Mfsd2b is expressed in whole hearts and isolated ACM of C57BL/6J mice and in human hearts.** (A) Relative gene expression of Mfsd2b in C57BL/6 heart tissue (n=7) and isolated ACM (n=5). (B) CT value of Mfsd2b in three different human hearts explanted during transplantation (left). Validation of amplicon size (103 bp) of qPCR product (right).

**SI Fig. 2: Blood pressure after AngII infusion by miniosmotic pumps in Mfsd2b^+/+^ and Mfsd2b^-/-^ mice over four weeks.** Serial systolic blood pressure measurements before and during AngII treatment. n_WT_=11, n_KO_=10. Data is presented as mean ± SEM. Mixed-effects analysis followed by Sidak's multiple comparison test was used for statistical analysis.

**SI Figure 3: Similar degree of fibrosis and heart failure gene expression in hearts of AngII-treated Mfsd2b^+/+^ and Mfsd2b^-/-^ mice.** (A) Representative histologic slices from Sirius Red staining of Mfsd2b^+/+^ (left) and Mfsd2b^-/-^ mice (right). (B) The average proportion of fibrosis in heart tissue from Mfsd2b^+/+^ and Mfsd2b^-/-^ mice was determined by Sirius Red staining. n_WT_=10, n_KO_=9. Data are presented as mean ± SEM. An unpaired Student t-test was used for statistical analysis. *p*-value is as indicated above the compared data sets. (C) Quantification of Nppa, NppB, Myh7, Postn, Acadm, CCN2 and Act1 gene expression by qRT-PCR analysis in hearts from Mfsd2b^+/+^ and Mfsd2b^-/-^ mice after AngII treatment. n_WT_=9, n_KO_=9. Data is presented as mean ± SEM. All data was tested by unpaired Student t-tests. *p*-values are as indicated above the compared data sets.

**SI Fig. 1: Mfsd2b is expressed in whole hearts and isolated ACM of C57BL/6J mice (A) and in human hearts (B).**

**
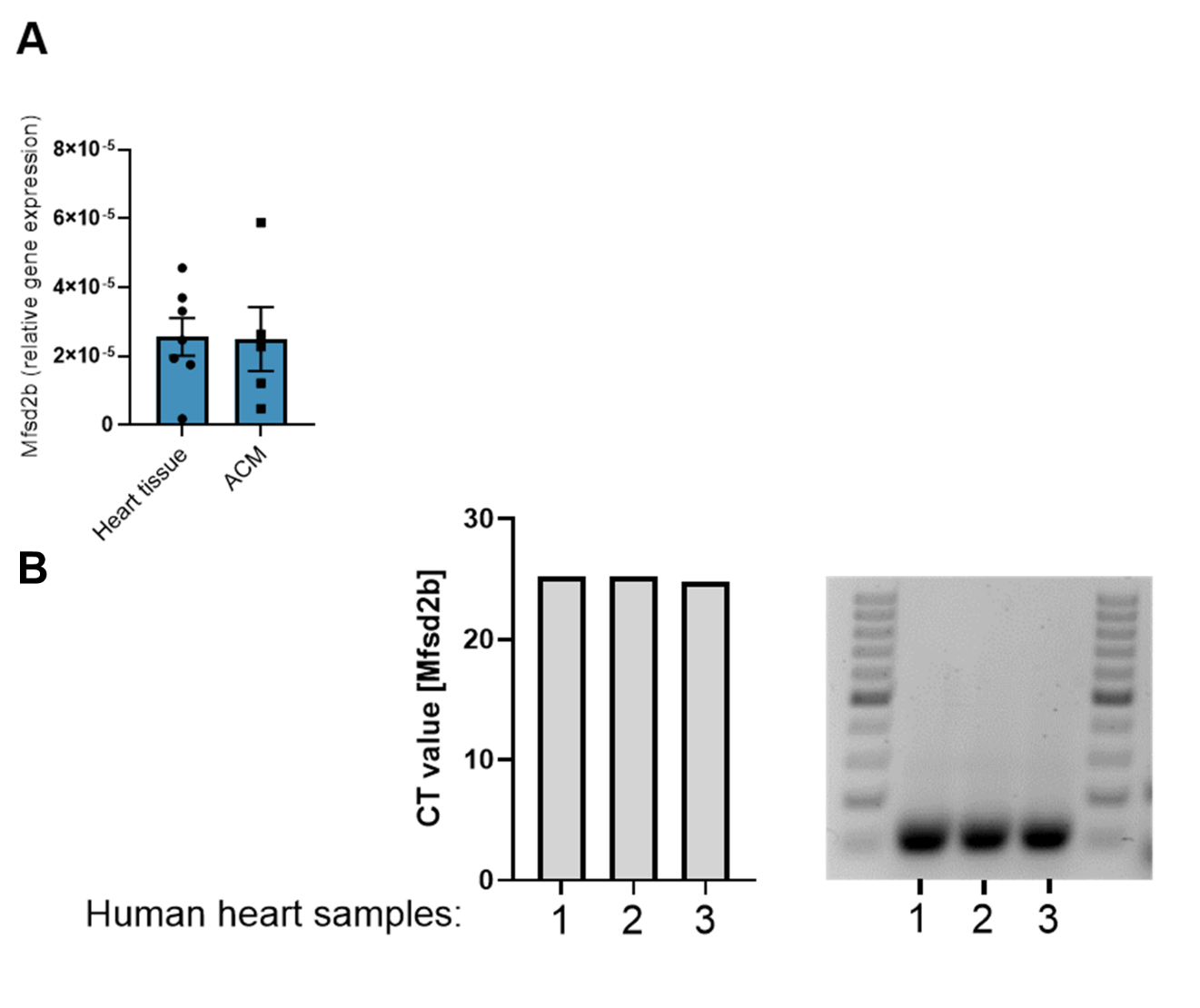
**

**SI Fig. 2: Blood pressure after AngII infusion by miniosmotic pumps in Mfsd2b^+/+^ and Mfsd2b^-/-^ mice over four weeks.**


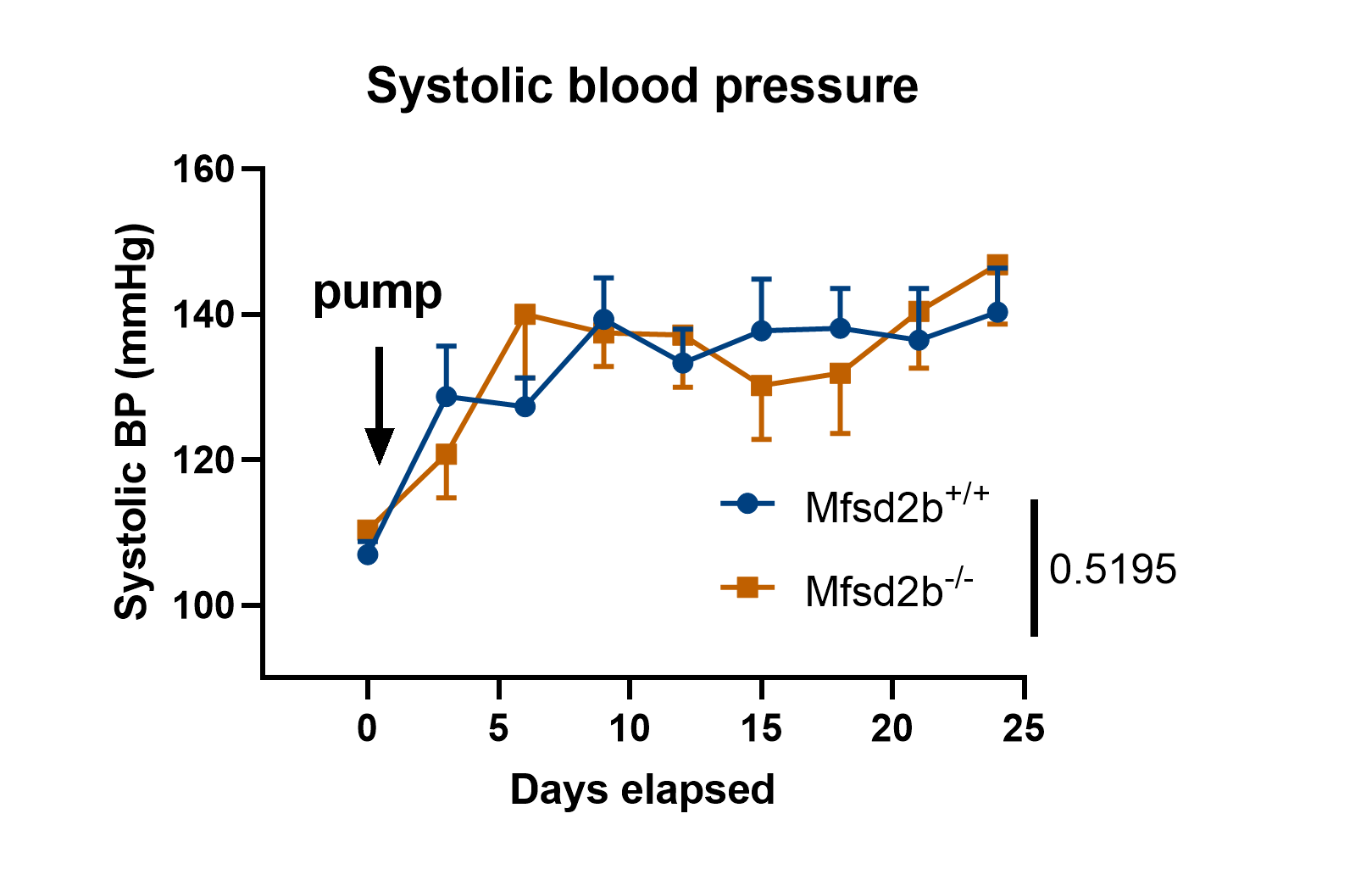


**SI Figure 3: Similar degree of fibrosis and heart failure genes following AngII-induced cardiac deterioration in Mfsd2b^+/+^ and Mfsd2b^-/-^ mice.**

**
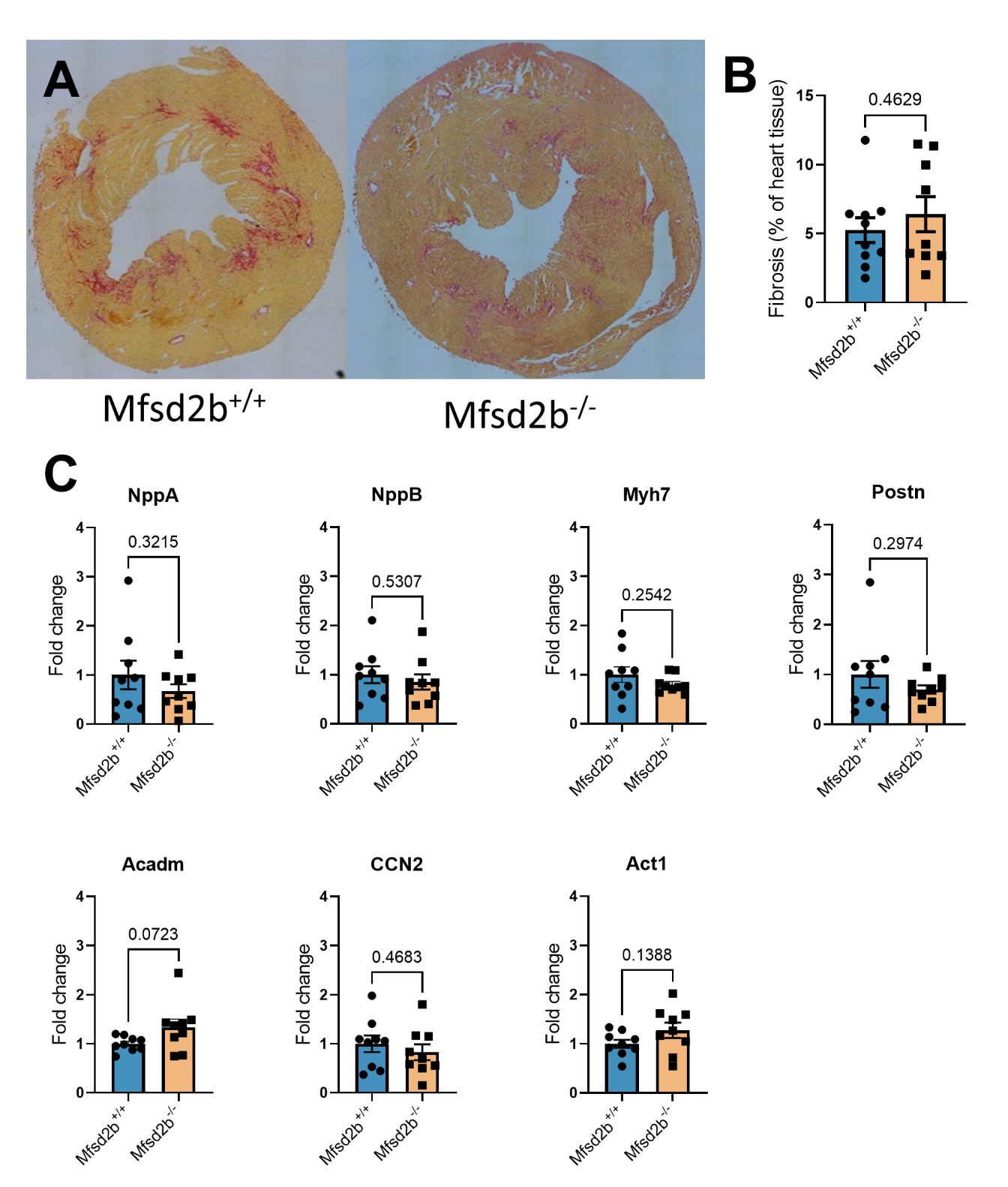
**

| **Time [min]** | **MeOH [%]** | **1 % aq. HCO_2_H [%]** | **Curve** |
| --- | --- | --- | --- |
| 0.00 | 10 | 90 | −2 |
| 3.00 | 100 | 0 | 0 |
| 8.00 | 100 | 0 | 0 |
| 8.01 | 10 | 0 | 0 |
| 10.0 | 10 | 90 | 0 |

**SI Table 1:** Gradient elution for S1P measurement via LCMS.

**SI Table 2:** Primer sequence used for qPCR.

| **Gene** | **Sequence5'-3'** | **Species** |
| --- | --- | --- |
| Mfsd2B | FW: CCTACCGGATGACTGTGGAG  RV: AGCCGCAATGCAGTAGAGA | mouse |
| NppA | FW: GTGCGGTGTCCAACACAGAT  RV: TCCAATCCTGTCAATCCTACCC | mouse |
| NppB | FW: GAGGTCACTCCTATCCTCTGG  RV: GCCATTTCCTCCGACTTTTCTC | mouse |
| Myh7 | FW: CCTGCGGAAGTCTGAGAAGG  RV: CTCGGGACACGATCTTGGC | mouse |
| PostN | FW: CCTGCCCTTATATGCTCTGCT  RV: AAACATGGTCAATAGGCATCACT | mouse |
| AcadM | FW: AGGGTTTAGTTTTGAGTTGACGG  RV: CCCCGCTTTTGTCATATTCCG | mouse |
| Ccn2 | FW: GGCCTCTTCTGCGATTTCG  RV: GCAGCTTGACCCTTCTCGG | mouse |
| Actc1 | FW: CTGGATTCTGGCGATGGTGTA  RV: CGGACAATTTCACGATCAGCA | mouse |
| LTCC | FW: TCCCGAGCACATCCCTACTC  RV: ACTGACGGTAGAGATGGTTGC | mouse |
| Mfsd2B | FW: GCCTTTGGGATCTTTGCGATG  RV: CAATGCTCACGCCAGATACAA | human |
